# Supplementary material for: Content-rich biological network constructed by mining PubMed abstracts
Source: BMC Bioinformatics. 2004 Oct 8;5:147. doi: 10.1186/1471-2105-5-147 (PMC528731; doi:10.1186/1471-2105-5-147)
Supplement: Additional File 2 — The original results of the above study (non-essential files are deleted to keep the file size under the limit set by BMC bioinformatics). [file 1471-2105-5-147-S2.bz2 › chilibotAdditionalFile2/dip05/338ID9228058E4140/html/TNFSF6_TNFRSF6.html]

 


 **TNFSF6** and **TNFRSF6** 
  
Found 4489 abstracts in PubMed, retrieved 05.  
 

 What does Google say? 
 PDF only 
| .edu only 

---

**Interactive relationship** (e.g. stimulation, inhibition, etc)

**Neutral relationship**- Apoptosis did not require prior exposure to interleukin 2, nor was it the consequence of CD178  [ **TNFSF6** ]  CD95  [ **TNFRSF6** ]  or TNF TNF R interactions, and was therefore clearly different from activation induced cell death.  Ref: 12893746 BloodBlood,
- Alterations in expression or shedding of membrane and soluble CD95L  [ **TNFSF6** ]  are associated with numerous diseases, and underscore the pathophysiological relevance of the CD95  [ **TNFRSF6** ]  CD95L  [ **TNFSF6** ]  system.  Ref: 12894217 Cell Death DifferCell Death Differ,

**Non-interactive relationship** (e.g. studied together, co-existance, homology, etc.)

- and expression of perforins, Fas  [ **TNFRSF6** ]  ligand  [ **TNFSF6** ] , and other functional markers.  Ref: 12891535 Gastroenterology, 2003
- While Fas  [ **TNFRSF6** ]  and FasL  [ **TNFSF6** ]  are involved in apoptosis of bystander cells, mitochondrial pathway is required for apoptosis of infected cells.  Ref: 12888173 Rev Med Interne, 2003
- Factors like cytokines, prostaglandine, microbes, MHC molecules, hormones, Fas  [ **TNFRSF6** ]  ligand  [ **TNFSF6** ] , heat shock proteins, phenotypes of dendritic cells and APCs, probably can be polarizing courses of eTLM pathway.  Ref: 12888311 Med Hypotheses, 2003
- CD95  [ **TNFRSF6** ]  ligand  [ **TNFSF6** ]  death factor and costimulatory molecule ? .  Ref: 12894217 Cell Death DifferCell Death Differ, 2003
- The CD95  [ **TNFRSF6** ]  ligand  [ **TNFSF6** ]  is involved as a death factor in the regulation of activation induced cell death, establishment of immune privilege and tumor cell survival.  Ref: 12894217 Cell Death DifferCell Death Differ, 2003
